# Role of Biochemical Markers in Invasive Ventilation of Coronavirus Disease 2019 Patients: Multinomial Regression and Survival Analysis

**DOI:** 10.7759/cureus.10054

**Published:** 2020-08-26

**Authors:** Muhammad Sohaib Asghar, Syed J Haider Kazmi, Noman A Khan, Mohammed Akram, Rumael Jawed, Wania Rafaey, Maira Hassan, Uzma Rasheed, Mehak Khan, Ali R Khan

**Affiliations:** 1 Internal Medicine, Dow International Medical College, Karachi, PAK; 2 Emergency Medicine, Liaquat National Hospital, Karachi, PAK; 3 General Surgery, Liaquat National Hospital, Karachi, PAK; 4 Internal Medicine, Liaquat National Hospital, Karachi, PAK; 5 Immunology, United Medical and Dental College, Karachi, PAK; 6 Infectious Diseases, United Medical and Dental College, Karachi, PAK

**Keywords:** invasive, non-invasive, ventilation, ventilator, mortality, covid-19, coronavirus, infectious diseases, pulmonology, pakistan

## Abstract

Background and objectives

Severe acute respiratory syndrome coronavirus 2 (SARS-CoV-2) is the pathogen responsible for the coronavirus disease 2019 (COVID-19) pandemic. The disease mainly affects the respiratory system of the patient, in particular, the lungs, which leads to patients presenting with acute respiratory distress syndrome and acute respiratory failure, with 5-15% of patients requiring observation in the intensive care unit (ICU) with respiratory support in the form of ventilation. This study was aimed at identifying the role of biochemical markers in the risk stratification of invasive and non-invasive ventilation of hospitalized COVID-19 patients.

Materials and methods

The study was conducted as a prospective, observational study of all admitted COVID-19 patients. A comparative analysis was performed of the survivors who were on invasive versus (vs) non-invasive ventilation and the non-survivors similarly. After computing the descriptive statistics, a multinomial logistic regression model was applied to obtain an unadjusted odds ratio (OR) at 95% confidence interval (CI), with Hosmer-Lemeshow (HL) goodness-of-fit test used to predict the fitness of the data. Kaplan-Meier survival curves were obtained for each of the laboratory investigations predicting survival along with the intensive care stay and invasive ventilation. A log-rank test was carried out to compare the survival distributions.

Results

A total of 373 included patients in the study had a mean age of 52.78 ± 15.76 years with females younger than males, and indifference amongst invasive vs non-invasively ventilated (p=0.821). Females were slightly more prone to invasive ventilation (p=0.097). Overall, 39% of the subjects did not need respiratory support, while 13% were on a ventilator, 16% on bilevel positive airway pressure/continuous positive airway pressure (BiPAP/CPAP), and 31% on supplemental oxygen therapy. Among the laboratory markers, mean hemoglobin was evidently lower in the invasive group, leukocytosis and thrombocytopenia were present in both invasively ventilated and non-surviving patients, while neutrophilia and lymphocytopenia were statistically indifferent among the mode of ventilation. Elevated urea, creatinine, and sodium were also significantly deranged laboratory markers amongst the invasively ventilated group. C-reactive protein (CRP) and lactate dehydrogenase (LDH) were elevated significantly in the invasive group, while serum ferritin was more frequently raised in the non-invasively ventilated group. Procalcitonin (PCT) was significantly associated with invasive ventilation as opposed to the non-invasive group. D-dimer was equally raised in both the groups at admission but significantly elevated in the invasive group at discharge. A multinomial regression model signified D-dimer (OR: 16.301), hypernatremia (OR: 12.738), creatinine (OR: 12.589), urea (OR: 12.576), and LDH (OR: 12.245) most significantly associated with death, while those for invasive ventilation were D-dimer (OR: 8.744), hypernatremia (OR: 4.532), PCT (OR: 3.829), neutrophilia (OR: 3.804), leukocytosis (OR: 3.330), and serum urea (OR: 3.312). Kaplan-Meier curves conclude total leucocyte count (TLC), neutrophils, lymphocytes, urea, creatinine, sodium, CRP, LDH, PCT, and D-dimer all significantly contributing to an early death.

Conclusion

The most significant marker for mortality was D-dimer, followed by serum sodium, urea/creatinine, LDH, ICU stay, and invasive ventilation.

## Introduction

The severe acute respiratory syndrome coronavirus 2 (SARS-CoV-2) is the pathogen responsible for the current coronavirus disease 2019 (COVID-19) pandemic. The disease mainly affects the respiratory system of the patients, particularly, the lungs, which leads to patients presenting with acute respiratory distress syndrome (ARDS) and acute respiratory failure [1]. Infection by the SARS-CoV-2 in the lungs leads to subpleural inflammation and increased vascular permeability, thus causing interstitial edema. Increased ventilation, tidal volume, and respiratory rate are physiological responses of the body to hypoxemia. Hypoxemia, along with increased metabolism from the inflammation, fever, and increased oxygen consumption further increases the respiratory rate. Patients often require careful observation as their condition can rapidly deteriorate, and the use of biomarkers is particularly helpful in evaluating the clinical status of the patients [2]. Studies have shown that 5-15% of patients with COVID-19 require observation in the intensive care unit (ICU), with respiratory support in the form of ventilation [1].

Invasive intubation through an endotracheal tube has commonly been used to treat patients during this pandemic. Till the end of February 2020, an estimated 3.2% of patients confirmed to have COVID-19 in China had been invasively intubated [3]. The current COVID-19 pandemic in Pakistan may prove to be difficult to handle, as the public and private hospitals in the country collectively lack the proper amount of ventilators required. Should there be a sudden increase in the number of COVID-19 cases, this along with factors within the healthcare system, such as lack of beds in the ICUs, will be crucial in managing a huge load of patients. The statistics so far in Pakistan in terms of the number of ventilators are also quite appalling, with only 1650 available for a population of 212.8 million people [4].

In an event that the cases of COVID-19 surge in the country, the ability of the healthcare system to adapt and counter this pandemic will be extremely difficult. This will lead to a shortage of ventilators due to the amount of severely ill patients requiring respiratory support [4]. Seeing that there is a considerable amount of evidence that proves that the use of protective ventilation (with low tidal volumes and pressures) will overall improve the clinical outcome of patients with ARDS, it is imperative that the use of ventilators must be appropriate in our patient population [5].

Keeping in mind the massive shortage of ventilators in the country, risk factors and biochemical markers should be identified in patients that hold a predictive value of the need for ventilation during the course of the disease of a patient, which will aid the healthcare system in managing the huge patient load. The risk factors, particularly the role of biomarkers that can effectively predict the severity of the disease and the need for ventilation among SARS-CoV-2 infected patients are not clear. This study aimed at identifying the role of biochemical markers in the risk stratification of invasive and non-invasive ventilation of hospitalized COVID-19 patients.

## Materials and methods

The study was conducted as a prospective, observational study including all the hospitalized COVID-19 patients (diagnosed via real-time polymerase chain reaction). The comparative analysis was performed among the survivors who were on invasive ventilation vs non-invasive ventilation and the non-survivors similarly. The statistical analysis for the laboratory investigations of all patients was conducted using Statistical Package for the Social Sciences (IBM SPSS Statistics for Windows, Version 25, Armonk, NY: IBM Corp.). The continuous variables were described as mean and standard deviation, and an independent sample t-test was used to compute the p-value. Categorical variables were described as frequency and percentages, and chi-square test or Fisher’s exact test was used according to the feasibility of the data presented. A p-value of <0.05 was considered statistically significant. All the highly significant values of <0.001 were rounded off as 0.001. A multinomial logistic regression model was applied to obtain an unadjusted odds ratio (OR) with a 95% confidence interval (CI), respectively for different cut-offs of laboratory values for an outcome of the disease. The Hosmer-Lemeshow (HL) goodness-of-fit test was used to predict the fitness of the logistic regression models for applicability to the categorical data. Kaplan-Meier survival curves were obtained for each of the laboratory investigations predicting survival along with the intensive care stay and invasive ventilation. A log-rank test was carried out to compare the survival distributions.

## Results

A total of 373 included patients in the study had a mean age of 52.78 ± 15.76 years with females younger than males, and indifference between invasive vs non-invasively ventilated (p=0.821). Approximately 50% of the study population belonged to the age group 50-75 years, with two-thirds of both invasive and non-invasive ventilation belonged to this age group (p=0.574). Females were slightly more prone to invasive ventilation (p=0.097). The length of hospital stay was also indifferent between the modes of ventilation, however, significant in survivors as compared to non-survivors (p=0.013). Overall, 39% of the subjects did not need respiratory support, while 13% were on a ventilator, 16% on bilevel positive airway pressure/continuous positive airway pressure (BiPAP/CPAP), and 31% on supplemental oxygen therapy, as shown in Table 1.

**Table 1:** Demographic data of the study population (n=373) * Indicates independent sample t-test used to compute the p-value. ** Chi-square test to compute the p-value. † Fisher’s exact test used to compute the p-value. Descriptive statistics are presented as Mean ± standard deviation. Frequencies are presented as n(%), where n= number of subjects. Abbreviations: BiPAP, bilevel positive airway pressure; CPAP, continuous positive airway pressure; ICU, intensive care unit; SD, standard deviation.

#	Variables	Characteristics						p-value
1	Age (in years)	Age group	<25	25-50		50-75	>75	-
		Total	15 (4.0%)	139 (37.3%)		204 (54.7%)	15 (4.0%)	
		Males	8 (3.2%)	89 (35.6%)		139 (55.6%)	14 (5.6%)	0.123^†^
		Females	7 (5.7%)	50 (40.6%)		65 (52.8%)	1 (0.8%)	
		Invasive ventilation	2 (4.0%)	13 (26.0%)		34 (68.0%)	1 (2.0%)	0.574^†^
		Non-invasive ventilation	2 (1.1%)	47 (26.7%)		122 (69.3%)	5 (2.8%)	
		Survivors	13 (4.8%)	122 (45.3%)		124 (46.1%)	10 (3.7%)	<0.001^†^
		Non-survivors	2 (1.9%)	18 (17.3%)		79 (76.0%)	5 (4.8%)	
2	Mean age (in years)	52.78 ± 15.76						-
		Males: 54.63 ± 15.22			Females: 48.85 ± 16.24			0.002*
		Invasive: 56.46 ± 13.78			Non-invasive: 56.94 ± 12.71			0.821*
		Survivors: 49.45 ± 16.03			Non-survivors: 61.02 ± 11.56			<0.001*
3	Gender	Males: 67.0% (n=250)			Females: 33.0% (n=123)			-
		Invasive: 19.0% (n=29)			Invasive: 28.8% (n=21)			0.097**
		Non-invasive: 81.0% (n=124)			Non-invasive: 71.2% (n=52)			
		Survivors: 72.8% (n=182)			Survivors: 70.7% (n=87)			0.675**
		Non-survivors: 27.2% (n=68)			Non-survivors: 29.3% (n=36)			
4	Hospital Stay	Ward: 64.3% (n=240)			ICU: 35.7% (n=133)			-
		Survivors: 88.8% (n=213)			Survivors: 42.1% (n=56)			<0.001**
		Non-survivors: 11.2% (n=27)			Non-survivors: 57.9% (n=77)			
5	Length of Hospital stay (in days)	8.09 ± 5.32						-
		Invasive: 8.46 ± 5.61			Non-invasive: 8.06 ± 5.48			0.658*
		Survivors: 8.52 ± 5.50			Non-survivors: 6.97 ± 4.56			0.013*
6	Mode of Respiration	Total: 60.6% (n=226)			No respirator: 39.4% (n=147)			-
		Invasive (On Ventilator): 13.4% (n=50)			Survivors: 18.0% (n=9)			<0.001**
					Non-survivors: 82.0% (n=41)			
		BiPAP/CPAP (Non-invasive): 15.8% (n=59)			Survivors: 50.8% (n=30)			
					Non-survivors: 49.2% (n=29)			
		Oxygen therapy (via mask/nasal cannula): 31.4% (n=117)			Survivors: 75.2% (n=88)			
					Non-survivors: 24.8% (n=29)			

**Figure 1:**
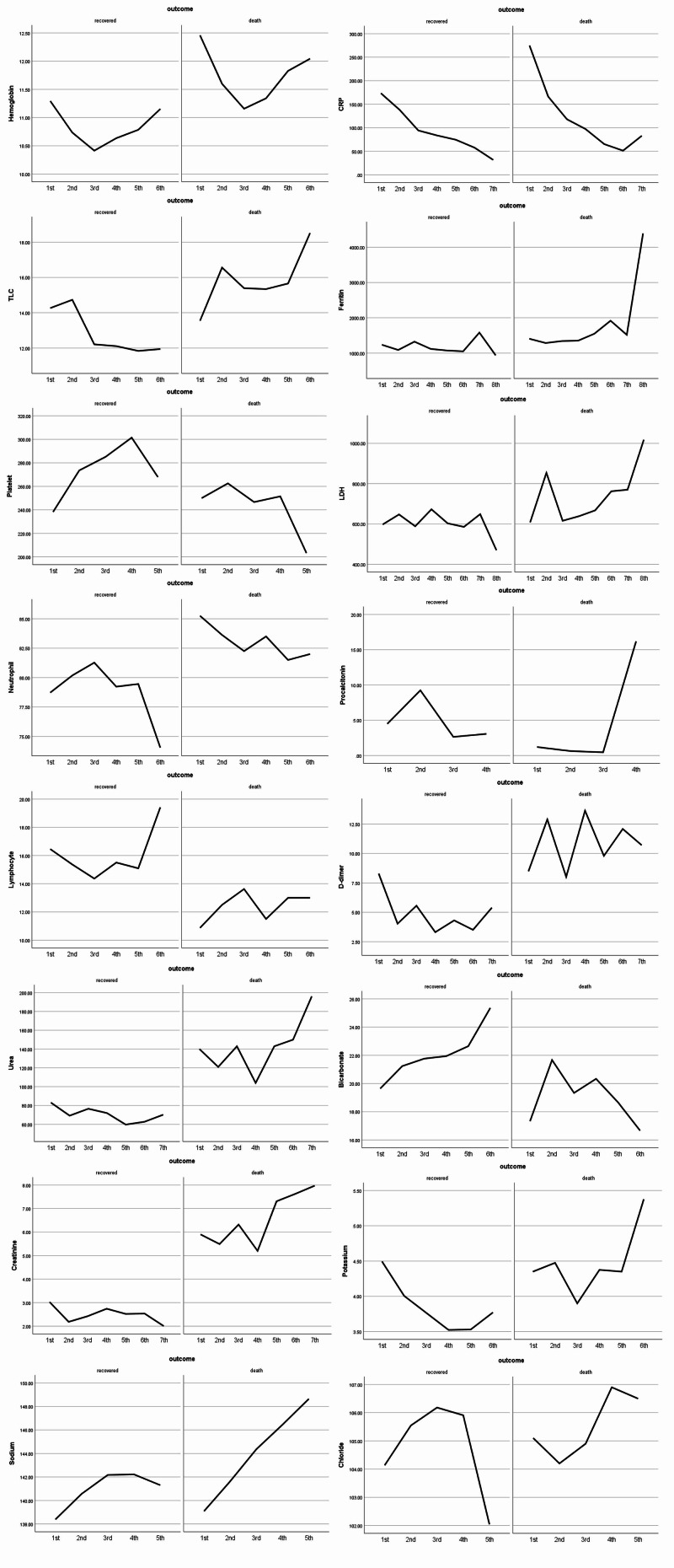
Graphical representation of in-hospital changes to the laboratory parameters on the follow-up intervals The y-axis shows varying mean values of laboratory markers, x-axis shows the number of follow-ups.

Now, coming to the biochemical markers, there were significant differences in the values of non-invasive group vs the invasive group, such as mean hemoglobin was evidently lower in the invasive group (p= 0.013), leukocytosis was a feature of 56% invasively ventilated and 38% non-invasively ventilated patients at admission and 72% vs 47% at discharge (p=0.003), thrombocytopenia was present (27%-28%) in both invasively ventilated and non-surviving patients respectively at discharge (p=0.009), while neutrophilia and lymphocytopenia were statistically indifferent between the modes of ventilation. Elevated urea (75% vs 53%), creatinine (64% vs 43%), and sodium (55% vs 25%) were also significantly deranged laboratory markers amongst the invasively ventilated group at discharge. C-reactive protein (CRP) levels (p=0.043) were elevated significantly in the invasive group at admission (74% vs 61%) while being insignificant at discharge. Lactate dehydrogenase (LDH) also significantly raised (p=0.022) in the invasively ventilated group at admission (81% vs 69%), while serum ferritin was more frequently raised in the non-invasively ventilated group (60% vs 68%) at admission. Procalcitonin (PCT) was significantly associated with invasive ventilation with 59% of patients had increased values at admission (p=0.005) and 64% at discharge (p=0.010) as opposed to 33% and 30% respectively in the non-invasive group. D-dimer was equally raised at admission in both the groups but significantly elevated (p=0.002) in the invasive group at discharge (94% vs 65%), as shown in Table 2. The in-hospital changes to all of these laboratory parameters along with their follow-ups are graphically represented in Figure 1.

**Table 2:** Comparison of laboratory investigations between the modes of ventilation of COVID-19 patients (n=373) Descriptive statistics are presented as mean ± standard deviation. Frequencies are presented as n (%), where n=number of subjects/total number of subjects. * Indicates independent sample t-test; ** indicates chi-square test; † indicates Fisher’s exact test. Abbreviations: COVID-19, coronavirus disease; TLC, total leukocyte count; CRP, C-reactive protein; LDH, lactate dehydrogenase;  PCT, procalcitonin.

Laboratory investigation		Invasive ventilation (n=50)	Non-invasive ventilation (n=176)	p-value	Survivors (n=269)	Non-survivors (n=104)	p-value
Hemoglobin	admission	11.70 ± 2.45	12.07 ± 2.28	0.315*	12.29 ± 2.31	11.65 ± 2.42	0.035*
	discharge	10.84 ± 2.14	11.75 ± 2.15	0.016*	11.66 ± 2.28	11.24 ± 2.22	0.176*
TLC	admission	13.92 ± 7.67	11.47 ± 6.70	0.044*	10.03 ± 5.62	14.17 ± 7.72	<0.001*
>11 x10^9^/L		n=28/50 (56.0%)	n=68/176 (38.6%)	0.028^**^	n=72/264 (27.3%)	n=58/104 (55.8%)	<0.001^**^
TLC	discharge	17.34 ± 13.62	12.89 ± 7.58	0.043*	9.79 ± 4.07	18.70 ± 11.60	<0.001*
>11 x10^9^/L		n=32/44 (72.7%)	n=70/147 (47.6%)	0.003^**^	n=51/148 (34.5%)	n=70/88 (79.5%)	<0.001^**^
Platelets	admission	238.79 ± 95.23	247.13 ± 122.03	0.659*	238.74 ± 120.17	236.67 ± 102.39	0.886*
<150 x10^9^/L		n=7/49 (14.3%)	n=31/172 (18.0%)	0.757^†^	n=50/253 (19.8%)	n=19/102 (18.6%)	0.970^**^
150-400		n=39/49 (79.6%)	n=126/172 (73.3%)		n=186/253 (73.5%)	n=76/102 (74.5%)	
>400 x10^9^/L		n=3/49 (6.1%)	n=15/172 (8.7%)		n=17/253 (6.7%)	n=7/102 (6.9%)	
Platelets	discharge	220.20 ± 124.93	281.86 ± 141.60	0.007*	276.79 ± 130.91	231.84 ± 145.43	0.020*
<150 x10^9^/L		n=12/44 (27.3%)	n=25/144 (17.4%)	0.009^**^	n=24/142 (16.9%)	n=25/89 (28.1%)	0.009^**^
150-400		n=31/44 (70.5%)	n=88/144 (61.1%)		n=90/142 (63.4%)	n=58/89 (65.2%)	
>400 x10^9^/L		n=1/44 (2.3%)	n=31/144 (21.5%)		n=28/142 (19.7%)	n=6/89 (6.7%)	
Neutrophil	admission	79.70 ± 9.13	75.68 ± 13.82	0.017*	72.98 ± 13.42	80.00 ± 12.03	<0.001*
>75 %		n=37/50 (74.0%)	n=108/176 (61.4%)	0.100^**^	n=109/264 (41.3%)	n=80/104 (76.9%)	<0.001^**^
Neutrophil	discharge	78.27 ± 12.41	74.40 ± 14.04	0.102*	70.52 ± 13.51	81.86 ± 10.34	<0.001*
>75 %		n=29/44 (65.9%)	n=81/146 (55.5%)	0.219^**^	n=66/146 (45.2%)	n=72/88 (81.8%)	<0.001^**^
Lymphocyte	admission	15.24 ± 8.82	17.46 ± 10.90	0.188*	20.15 ± 11.19	14.14 ± 9.45	<0.001*
<20 %		n=38/50 (76.0%)	n=125/176 (71.0%)	0.489^**^	n=136/264 (51.5%)	n=85/104 (81.7%)	<0.001^**^
Lymphocyte	discharge	15.59 ± 11.50	18.47 ± 12.16	0.165*	22.15 ± 12.49	12.37 ± 8.11	<0.001*
<20 %		n=34/44 (77.3%)	n=98/146 (67.1%)	0.200**	n=65/146 (44.5%)	n=73/88 (83.0%)	<0.001^**^
Urea	admission	59.72 ± 52.33	54.64 ± 47.88	0.522*	43.22 ± 42.67	69.98 ± 52.08	<0.001*
>49 mg/dL		n=20/49 (40.8%)	n=63/174 (36.2%)	0.555^**^	n=51/257 (19.8%)	n=56/103 (54.4%)	<0.001^**^
Urea	discharge	129.89 ± 76.10	80.08 ± 68.09	0.001*	46.39 ± 37.97	138.71 ± 73.83	<0.001*
>49 mg/dL		n=27/36 (75.0%)	n=67/125 (53.6%)	0.022^**^	n=39/122 (32.0%)	n=65/76 (85.5%)	<0.001^**^
Creatinine	admission	2.12 ± 3.35	1.73 ± 2.61	0.380*	1.51 ± 2.65	2.19 ± 2.71	0.046*
>1.3 mg/dL		n=20/50 (40.0%)	n=62/174 (35.6%)	0.572^**^	n=57/258 (22.1%)	n=54/104 (51.9%)	0.001^**^
Creatinine	discharge	3.16 ± 2.57	2.03 ± 2.12	0.019*	1.33 ± 1.54	3.27 ± 2.43	<0.001*
>1.3 mg/dL		n=23/36 (63.9%)	n=54/125 (43.2%)	0.029^**^	n=28/122 (23.0%)	n=60/76 (78.9%)	0.001^**^
Sodium	admission	138.78 ± 6.78	138.35 ± 7.28	0.713*	138.09 ± 5.44	139.25 ± 8.54	0.174*
>145 mEq/L		n=9/50 (18.0%)	n=12/174 (6.9%)	0.026^†^	n=7/255 (2.7%)	n=16/104 (15.4%)	<0.001^**^
Sodium	discharge	146.63 ± 6.59	142.31 ± 8.37	0.002*	139.15 ± 4.59	147.77 ± 9.16	<0.001*
>145 mEq/L		n=20/36 (55.6%)	n=31/124 (25.0%)	0.001^**^	n=13/121 (10.7%)	n=46/76 (60.5%)	<0.001^**^
Potassium	admission	4.19 ± 1.13	4.12 ± 0.85	0.671*	4.19 ± 0.93	4.05 ± 0.83	0.227*
	discharge	4.27 ± 1.21	4.02 ± 0.89	0.176*	3.83 ± 0.63	4.35 ± 1.23	0.001*
Chloride	admission	102.92 ± 6.97	102.55 ± 6.32	0.722*	103.14 ± 5.47	102.50 ± 7.23	0.408*
	discharge	105.58 ± 7.06	103.31 ± 7.38	0.103*	101.87 ± 5.40	106.59 ± 8.22	<0.001*
Bicarbonate	admission	19.58 ± 4.22	19.75 ± 4.04	0.794*	20.36 ± 3.51	19.04 ± 4.31	0.010*
	discharge	22.00 ± 6.08	21.93 ± 4.65	0.946*	22.85 ± 4.14	20.59 ± 5.23	0.002*
CRP	admission	193.70 ± 116.32	153.96 ± 120.63	0.043*	125.23 ± 107.72	201.51 ± 120.22	<0.001*
>100 mg/L		n=35/47 (74.5%)	n=104/169 (61.5%)	0.102^**^	n=94/204 (46.1%)	n=76/98 (77.6%)	<0.001^**^
CRP	discharge	93.35 ± 103.55	82.43 ± 107.50	0.576*	56.38 ± 84.66	129.03 ± 120.00	<0.001*
>50 mg/L		n=17/39 (43.6%)	n=49/128 (38.3%)	0.553^**^	n=37/125 (29.6%)	n=43/77 (55.8%)	<0.001^**^
LDH	admission	988.67 ± 1930.65	602.53 ± 372.77	0.022*	519.36 ± 267.69	881.12 ± 1398.56	0.019*
>400 U/L		n=35/43 (81.4%)	n=103/148 (69.6%)	0.128^**^	n=101/178 (56.7%)	n=71/87 (81.6%)	<0.001^**^
LDH	discharge	1080.32 ± 1691.70	823.69 ± 1204.86	0.342*	482.82 ± 347.10	1319.46 ± 1803.44	0.001*
>400 U/L		n=28/34 (82.4%)	n=66/96 (68.8%)	0.128^**^	n=52/97 (53.6%)	n=59/63 (93.7%)	<0.001^**^
Ferritin	admission	2816.83 ± 7410.90	1508.93 ± 2372.26	0.061*	1177.76 ± 1610.68	2451.05 ± 5652.39	0.041*
>500 ng/mL		n=26/43 (60.5%)	n=106/155 (68.4%)	0.330^**^	n=104/185 (56.2%)	n=60/89 (67.4%)	0.077^**^
Ferritin	discharge	2874.63 ± 4060.67	2528.16 ± 4945.65	0.719*	1102.14 ± 1452.94	4035.69 ± 6143.00	<0.001*
>500 ng/mL		n=25/33 (75.8%)	n=72/92 (78.3%)	0.767^**^	n=60/90 (66.7%)	n=54/63 (85.7%)	0.008^**^
PCT	admission	4.33 ± 10.72	3.17 ± 11.23	0.578*	1.79 ± 8.93	4.81 ± 12.09	0.742*
>0.5 ng/mL		n=23/39 (59.0%)	n=35/106 (33.0%)	0.005^**^	n=22/95 (23.2%)	n=43/78 (55.1%)	<0.001^**^
PCT	discharge	13.05 ± 25.08	7.90 ± 22.76	0.407*	2.95 ± 12.32	14.27 ± 27.73	0.078*
>0.5 ng/mL		n=16/25 (64.0%)	n=11/36 (30.6%)	0.010^**^	n=7/29 (24.1%)	n=22/37 (59.5%)	0.004^**^
D-dimer	admission	9.17 ± 11.99	7.09 ± 12.55	0.358*	4.47 ± 8.92	11.45 ± 15.89	<0.001*
>1.0 mcg/mL		n=30/40 (75.7%)	n=85/123 (69.1%)	0.477^**^	n=76/136 (55.9%)	n=71/83 (85.5%)	<0.001^**^
D-dimer	discharge	13.59 ± 18.14	7.70 ± 11.77	0.036*	4.10 ± 7.30	14.70 ± 16.90	<0.001*
>1.0 mcg/mL		n=31/33 (93.9%)	n=61/93 (65.6%)	0.002^**^	n=51/94 (54.3%)	n=57/61 (93.4%)	<0.001^**^

Table 3 shows multinomial regression model for the invasive ventilation along with the laboratory values of non-surviving patients showing thrombocytopenia (OR: 1.815, CI: 0.956-3.449), leukocytosis (OR: 7.397, CI: 3.983-13.736), neutrophilia (OR: 5.455, CI: 2.899-10.264), lymphocytopenia (OR: 7.652, CI: 3.570-16.402), elevated urea (OR: 12.576, CI: 5.978-26.456), deranged creatinine (OR: 12.589, CI: 6.287-25.210), hypernatremia (OR: 12.738, CI: 6.098-26.609), elevated CRP level (OR: 3.893, CI: 2.071-7.315), LDH (OR: 12.245, CI: 4.122-36.373), ferritin (OR: 3.000, CI: 1.307-6.885), procalcitonin (OR: 4.610, CI: 1.574-13.496) and D-dimer (OR: 16.301, CI: 4.768-55.734), all significantly associated with the outcome of the disease as death. The stay in the intensive care unit (OR: 10.847, 6.397-18.392) and being on a ventilator (OR: 9.359, CI: 3.493-25.078) were also significant factors linked with mortality. The most significant laboratory factors for invasive ventilation were found to be D-dimer (OR: 8.744, CI: 1.997-55.734), followed by serum sodium (OR: 4.532, CI: 2.128-9.650), procalcitonin (OR: 3.829, CI: 1.342-10.927), neutrophilia (OR: 3.804, CI: 1.839-7.869), leukocytosis (OR: 3.330, CI: 1.590-6.971), and serum urea (OR: 3.312, CI: 1.466-7.482).

**Table 3:** Multinomial regression of COVID-19 patients for invasive ventilation and survival (n=373) Abbreviations: COVID-19, coronavirus disease; ICU, intensive care unit; BiPAP, bilevel positive airway pressure; CPAP, continuous positive airway pressure; S.E., standard error; CRP, C-reactive protein; LDH, lactate dehydrogenase; PCT, procalcitonin; TLC, total leukocyte count.

#	Variable state		Unadjusted odds ratio (OR)	S.E	95% confidence interval	Wald	p-value
1	ICU	Hospital stay	10.847	0.269	6.397–18.392	78.304	<0.001
	Invasive ventilation	Ventilator	9.359	0.503	3.493–25.078	19.777	<0.001
	Non-invasive ventilation	BiPAP/CPAP	0.341	0.337	0.176–0.660	10.189	0.001
		Oxygen mask	0.072	0.426	0.031–0.167	38.037	<0.001
2	Hemoglobin (<12 g/dL)	Ventilator	2.263	0.361	1.116–4.589	5.125	0.024
		Death	1.525	0.274	0.891–2.609	2.367	0.124
	TLC (>11 x 10^9^/L)	Ventilator	3.330	0.377	1.590–6.971	10.182	0.001
		Death	7.397	0.316	3.983–13.736	40.137	<0.001
	Platelet count (<150 x 10^9^/L)	Ventilator	1.520	0.385	0.715–3.234	1.183	0.277
		Death	1.815	0.327	0.956–3.449	3.317	0.069
	Neutrophils (>75%)	Ventilator	3.804	0.371	1.839–7.869	12.972	<0.001
		Death	5.535	0.282	3.183–9.627	36.728	<0.001
	Lymphocytes (<20%)	Ventilator	2.336	0.350	1.176–4.639	5.876	0.015
		Death	7.652	0.389	3.570–16.402	27.372	<0.001
3	Urea (>49 mg/dL)	Ventilator	3.312	0.416	1.466–7.482	8.293	0.004
		Death	12.576	0.379	5.978–26.456	44.518	<0.001
	Creatinine (>1.3 mg/dL)	Ventilator	2.640	0.382	1.248–5.585	6.452	0.011
		Death	12.589	0.354	6.287–25.210	51.110	<0.001
	Sodium (>145 mEq/L)	Ventilator	4.532	0.386	2.128–9.650	15.352	<0.001
		Death	12.738	0.376	6.098–26.609	45.840	<0.001
4	CRP (>100 mg/L)	Ventilator	2.909	0.367	1.418–5.968	8.483	0.004
		Death	3.964	0.280	2.290–6.860	24.218	<0.001
	LDH (>400 U/L)	Ventilator	2.714	0.415	1.202–6.128	5.776	0.016
		Death	12.245	0.555	4.122–36.373	20.340	<0.001
	Ferritin (>500 ng/mL)	Ventilator	1.088	0.457	0.445–2.664	0.034	0.853
		Death	3.000	0.424	1.307–6.885	6.719	0.010
	PCT (>0.5 ng/mL)	Ventilator	3.829	0.535	1.342–10.927	6.297	0.012
		Death	4.610	0.548	1.574–13.496	7.773	0.005
	D-dimer (>1.0 mcg/mL)	Ventilator	8.744	0.754	1.997–38.291	8.280	0.004
		Death	16.301	0.627	4.768–55.734	19.802	<0.001

**Figure 2:**
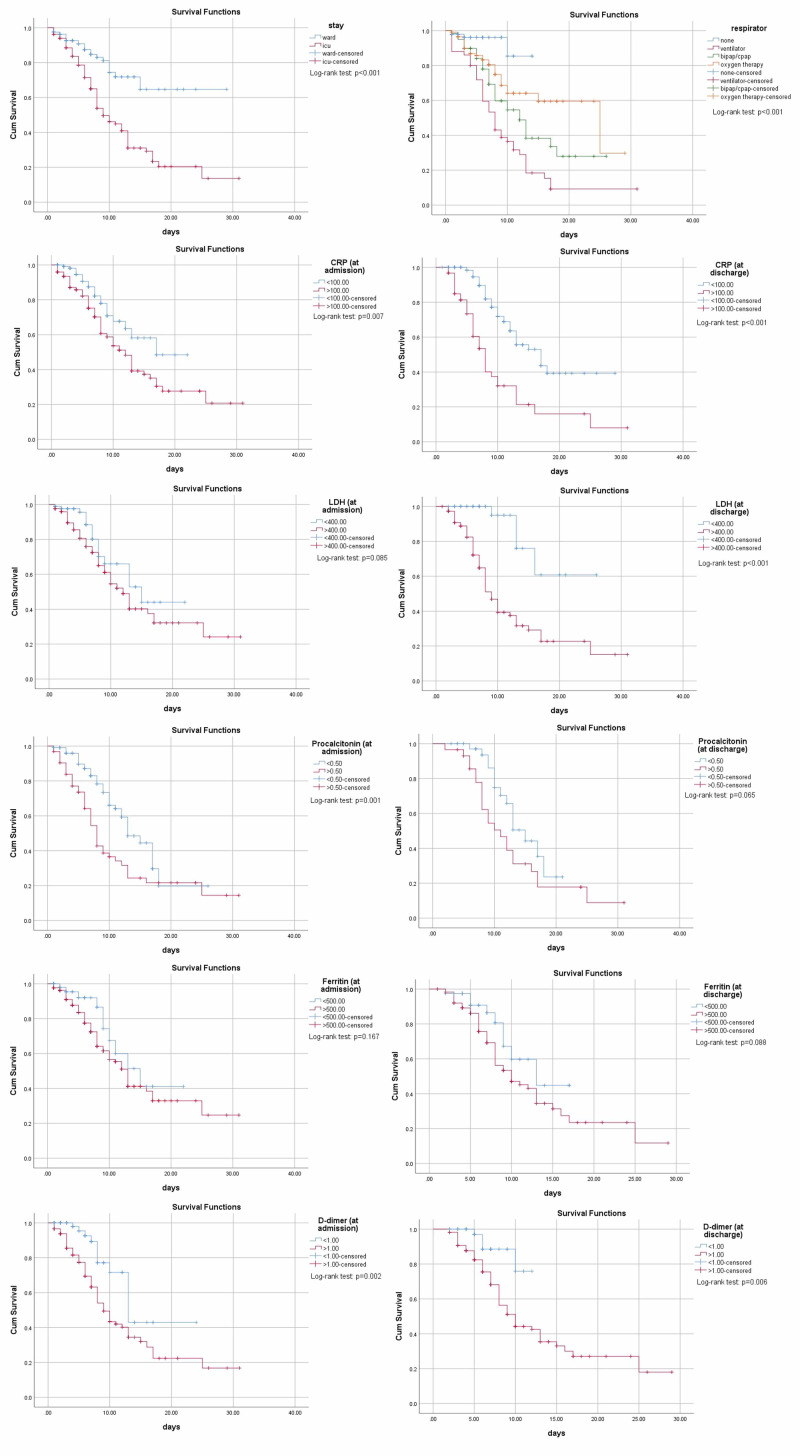
Kaplan-Meier curves for ICU, mode of ventilation, CRP, LDH, procalcitonin, ferritin, and D-dimer (at admission and discharge respectively) Abbreviations: ICU, intensive care unit; BiPAP, bilevel positive airway pressure; CPAP, Continuous positive airway pressure; CRP, C-reactive protein; LDH, lactate dehydrogenase.

**Figure 3:**
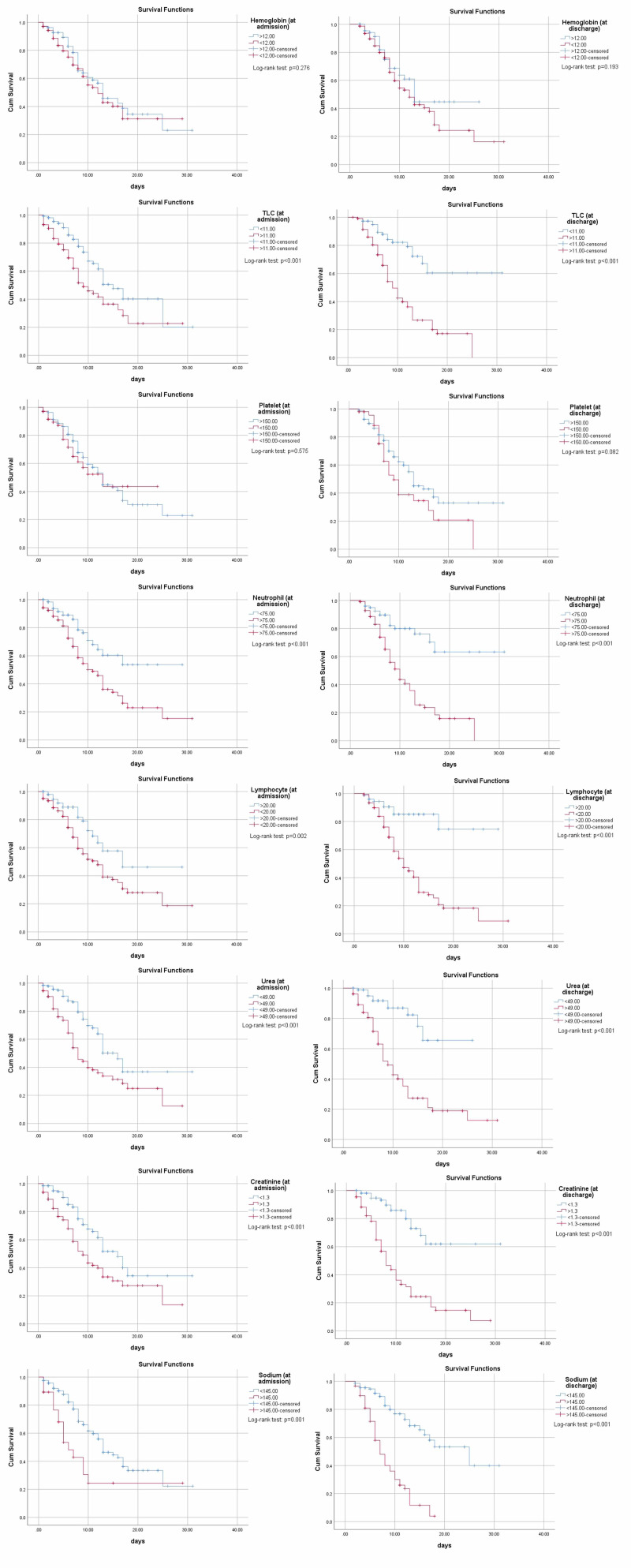
Kaplan-Meier curves for hemoglobin, TLC, platelet, neutrophil, lymphocyte, urea, creatinine, and sodium of COVID-19 patients (both at admission and discharge respectively) Abbreviation: TLC, total leukocyte count.

All the above markers were utilized for obtaining Kaplan-Meier curves to conclude the survival analysis, showing ICU stay, being on a ventilator, increased total leukocyte count (TLC), neutrophil count, decreased lymphocytes, deranged urea, creatinine, elevated sodium, CRP, LDH, PCT, and D-dimer all significantly contributing to early death as shown in Figure 2 and Figure 3.

## Discussion

This study established the role of important biochemical markers in predicting the need for invasive ventilation. We found that D-dimer, CRP, LDH, procalcitonin, sodium, urea, and creatinine all had significantly vital roles in the invasive ventilation of COVID-19 patients. Overall, our study showed that 13.4% of patients required invasive ventilation. This percentage falls in the range of previously conducted various studies in Pakistan, China, and the United States of America [6-8].

A study conducted by Hur, et al. found that the median age of their intubated patients was higher than the median age of patients that did not require intubation (65 vs 57 years). Age was a significant factor in determining whether the patients required invasive ventilation or not [9]. Our results showed that 67.3% of intubated patients were in the age range of 50-75 years (with a mean age of 56.46 ± 13.78), however, the age of patients on non-invasive was not significantly different from those on invasive ventilation. Advancing age did play a role in the mortality of our study population, with non-survivors having a higher mean age than survivors. This finding coincides with an earlier study on a similar population [6]. A high mortality rate was also seen in the invasively ventilated patients (82%) in our study which is much higher than what has been reported in Italy (23.3%) and the United States of America (15.2%) [9,10].

Our study found that D-dimer was the most significant biomarker associated with mortality and was an effective biomarker in predicting the need for mechanical ventilation in COVID-19 patients. This finding is similar to the results from a nationwide study conducted in China, where they reported elevated D-dimer on admission suggested a greater likelihood of invasive mechanical ventilation. The percentage of patients in the invasive and non-invasively ventilation groups with elevated D-dimer levels on admission were slightly different from our study, with a higher percentage of our subjects having elevated D-dimer levels (75.7% vs 66.7% in the invasive group and 69.1% vs 44.6% in the non-invasive group respectively) [11].

LDH was also found to be significantly elevated on admission in patients that required invasive ventilation and was the second most effective predictor of mortality after D-dimer. Studies conducted in China and Korea support our results of LDH as an effective predictor of the need for invasive ventilation in COVID-19 patients [11,12]. When looking at the elevated levels of LDH on admission in the invasive and non-invasive groups, the percentage of patients in our study was 81.4% and 69.6% respectively. This is similar to elevated LDH seen in China (97.2% vs 82.8% in the invasive and non-invasive groups, respectively) [11].

Our study showed that CRP was associated with invasive ventilation on admission, but showed no significant association on discharge. This finding is supported by the results of a study conducted by Herold, et al. who strongly associated CRP with invasive ventilation, but as the disease progressed the predictive value of CRP for the need for respiratory support did not improve [13]. The mean CRP in our study for invasively ventilated patients (on admission) was 193.70 ± 116.32, which was much higher than the study conducted in Wuhan, China where the mean CRP was noted to be 116.1 ± 94.2 [14]. Serum ferritin was not significantly associated with invasive ventilation among our patients. In fact, elevated serum ferritin was more of a feature of non-invasively ventilated patients. A study conducted to see the association between the iron profile and the hypoxemic respiratory failure in COVID-19 patients also did not find a significant link between serum ferritin and hypoxemia, thus the need for ventilation [15].

Serum urea, creatinine, and sodium levels were also associated with invasive ventilation. This may be due to the fact that COVID-19 patients often develop acute kidney injury (AKI) after hospitalization and this is accompanied by a high mortality rate [1]. Another study suggested high urea and creatinine with severe patients [16], while our results also showing urea (75% vs 53%) and creatinine (64% vs 43%) in the invasive vs non-invasively ventilated groups, respectively. Thrombocytopenia was present slightly more in non-survived patients in our results, like previous studies suggesting consumptive coagulopathy in deceased patients leading to thrombocytopenia but thrombocytosis in survived patients [6]. A meta-analysis conducted on the association of biochemical markers with the severity of the disease concluded CRP, PCT, and serum ferritin found more elevated in the severe vs non-severe group, a finding synchronizing with our results [17].

## Conclusions

The most significant marker for mortality was D-dimer followed by serum sodium, LDH, urea/creatinine, ICU stay, and invasive ventilation respectively. Decreased survival was also associated with various deranged laboratory markers including raised total leukocyte count (TLC), high neutrophil count, decreased lymphocytes, deranged urea, creatinine, elevated sodium, CRP, LDH, PCT, and D-dimer. Serum urea, creatinine, and sodium were significantly increased in patients with invasive ventilation at discharge, as compared to admitting values being similar in both the invasive and non-invasively ventilated groups, indicating that being on a ventilator also puts an increased risk of acute kidney injuries and electrolytes imbalance.
